# Short Chain Fatty Acids Prevent High-fat-diet-induced Obesity in Mice by Regulating G Protein-coupled Receptors and Gut Microbiota

**DOI:** 10.1038/srep37589

**Published:** 2016-11-28

**Authors:** Yuanyuan Lu, Chaonan Fan, Ping Li, Yanfei Lu, Xuelian Chang, Kemin Qi

**Affiliations:** 1 Nutrition Research Unit, Beijing Pediatric Research Institute, Beijing Children’s Hospital, Capital Medical University, Beijing 100045, China

## Abstract

Elucidating the mechanisms by which short chain fatty acids (SCFA) reduce body weight may assist in the development of an effective weight control strategy. Dietary supplementation of acetate, propionate, butyrate or their admixture was shown to significantly inhibit the body weight gain induced by high-fat diet feeding. Supplementation of SCFAs caused significant changes in the expressions of G-protein coupled receptor 43 (GPR43) and GPR41 characterized by increases in the adipose tissue and reductions in the colon. Additionally, they influenced the bacterial community structure in feces, with a reduction in the proportion of Firmicutes and an increase in the proportion of Bacteroidetes. The effects of dietary SCFAs on the GPR expression and gut microbiota composition may further result in body weight reduction by enhancing triglyceride hydrolysis and FFA oxidation in the adipose tissue, promoting beige adipogenesis and mitochondrial biogenesis, and inhibiting chronic inflammation.

The prevalence of obesity has risen substantially worldwide in both children and adults during the past several decades. Obesity and its associated complications, especially non-communicable chronic diseases, impose a double burden in health and costs^1^,^2^. Thus, improved strategies for obesity prevention and control are urgently needed. Epidemiological and experimental studies have demonstrated that a generous intake of dietary fiber reduces the risk for developing many chronic diseases, such as cardiovascular diseases, diabetes, certain gastrointestinal disorders, and obesity^3^,^4^,^5^,^6^. Feeding rodents a high level of dietary fiber protects against the high-fat-diet-induced increases in body weight and fat mass. Moreover, a higher intake of dietary fiber can inhibit appetite and decrease body weight in humans^7^,^8^. Emerging evidence has shown that these effects may be partly mediated by the short chain fatty acids (SCFAs) produced via fermentation of indigestible dietary carbohydrates (predominantly resistant starch and dietary fiber) by the intestinal microbiota^3^,^6^. However, large amounts of dietary fibers are generally not well tolerated as they are associated with gastrointestinal side effects. Thus, SCFAs have been administered in clinical trials, and the results showed that SCFAs have a positive effect on the management of ulcerative colitis, Crohn’s disease, diarrhea and obesity^9^,^10^. Targeting the mechanisms by which SCFAs reduce body weight may provide a more effective approach to weight control.

The major SCFAs produced by bacterial fermentation are acetate, propionate and butyrate, with an approximate molar ratio of 60:20:20, respectively, in the colon and stool^11^. Acetate and propionate are predominantly produced by bacterial species belonging to the Bacteroidetes phylum, whereas butyrate is primarily produced by the Firmicutes phylum^12^. SCFAs have been shown to mediate a variety of biological activities by targeting multiple organs and tissue sites. Numerous physiological and clinical studies have demonstrated that SCFAs are also a significant source of calories and have trophic effects on both the small and large bowel. Meanwhile, SCFAs may play beneficial roles in appetite regulation and lipid and glucose metabolism by epigenetically regulating related genes^13^,^14^. SCFAs also act as signal transduction molecules and bind and activate orphan G-protein-coupled receptors, such as GPR43 and GPR41, which are also known as free fatty acid receptor 2 and 3 (FFAR2 and FFAR3), respectively. GPR43 and GPR41 are both widely expressed in the small intestine and colon, as well as tissues and organs beyond the gut^15^,^16^. However, there is some debate regarding the role of these receptors in the regulation of energy balance *in vivo*. Studies have shown that genetically modified mice deficient in GPR43 are obese on a normal diet, whereas mice overexpressing GPR43 specifically in adipose tissue are protected against diet-induced obesity^17^. Inconsistently, high-fat diet (HFD)-fed GPR43-deficient mice were shown to have lower body fat mass and increased lean body mass accompanied by improved insulin sensitivity and increased energy expenditure compared to those of the normal diet-fed mice^18^. Thus, the protective or causative effects of GPR43 in obesity need to be clarified^16^.

After Bäckhed and his coworkers found that gut microbiota, acting as an environmental factor, regulate fat storage^19^, a considerable body of studies has shown alterations in the composition of the intestinal microbiota, primarily consisting of changes in the relative abundance of the phyla Firmicutes in obesity^20^,^21^, which further leads to changes in SCFA production. The fecal SCFA concentration was shown to be increased in genetically obese (ob/ob) mice and obese humans^22^,^23^, whereas it was decreased in high-fat diet-induced obese (DIO) mice^17^. Furthermore, germ-free mice with very low gut SCFA levels were protected from diet-induced obesity^19^. Recently, application of exogenous acetate, propionate or butyrate has been confirmed to prevent weight gain in DIO mice and overweight humans^24^,^25^. Therefore, the interactions between gut microbiota, SCFAs and their receptors, and host energy metabolism are shown to be complex by the contradictory reports in obesity. In this study, using a high-fat DIO mouse model, we determined the effects of exogenous SCFA application on body weight gain and associated changes in the GPR expressions, gut microbiota and fat oxidation.

## Results

### SCFAs inhibit body weight gain in DIO mice

**Figure 1:**
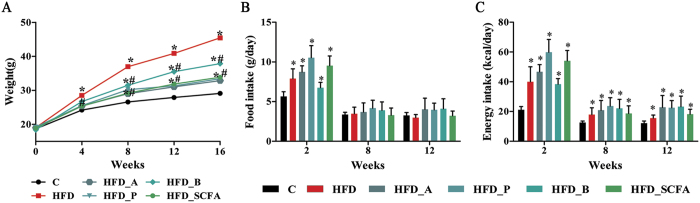
SCFAs protect against the high-fat diet induced increases in body weight. Three- to four-week-old C57BL/6 J male mice were fed a HFD diet and four SCFA-containing HFD diets—HFD-A, HFD-P, HFD-B and HFD-SCFA—with a low-fat diet as the control (**C**). (**A**) Body weight changes during the sixteen weeks of feeding (n = 15). (**B** and **C**) Food and energy intake per mouse at 2, 8 and 12 weeks of feeding (n = 15). Data are shown as the mean ± SD. *Compared to the lean control (**C**) group, P < 0.05; #compared to the HFD group, P < 0.05.

As shown in Fig. 1, mice administered the HFD gained weight steadily over time and showed an increase in body weight that was 56.12% higher than that of the control diet group after 16 weeks of feeding (P < 0.05). Dietary supplementation of acetate, propionate, butyrate or their admixture significantly suppressed the HFD-induced weight gain, with acetate having the greatest effects at 72.31% (P < 0.05) (Fig. 1A). Two weeks after the feeding intervention, both food and energy intake were increased in the HFD-fed mice (P < 0.05), and thereafter, energy intake remained higher (P < 0.05) with no differences in food intake at both 8 and 12 weeks compared to that of the control lean mice (Fig. 1B and C). Dietary supplementation of acetate, propionate, butyrate or their admixture did not change the intakes of both food and energy at 8 and 12 weeks after the feeding intervention (P > 0.05) (Fig. 1B and C).

### SCFAs affect biochemical parameters and fecal SCFA excretion in DIO mice

The effects of dietary SCFA supplementation on biochemical parameters are shown in Table 1. Plasma concentrations of glucose, triglycerides, cholesterol, insulin, IL-1β, IL-6 and MCP-1 were increased, and free fatty acids were decreased in the HFD-fed mice compared to those of the lean control mice (P < 0.05). Dietary supplementation of acetate, propionate, butyrate or their admixture generally attenuated the increases in triglycerides, cholesterol, IL-1β, IL-6 or MCP-1 (P < 0.05) promoted by the HFD with no effects on plasma glucose and insulin. Fecal propionate or butyrate concentration was increased in the HFD-P- and HFD-B-fed mice, respectively, compared to that of the HFD-fed mice (P < 0.05), whereas no changes in the levels of SCFAs were observed in the HFD-A-fed mice (P > 0.05), suggesting that dietary acetate was more efficiently absorbed by the intestine.

### SCFAs affect the GPR expression in DIO mice

**Figure 2:**
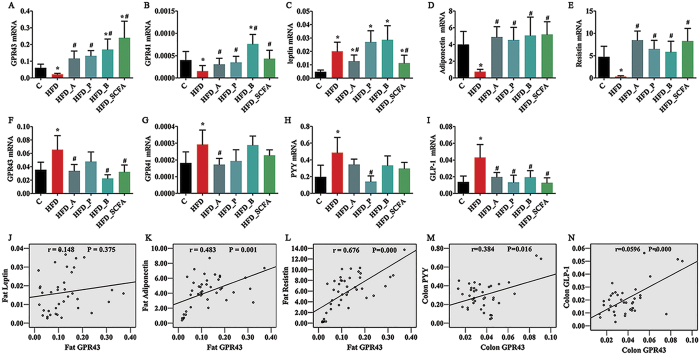
SCFAs regulate mRNA expressions of GRP43, GPR41, gut hormone peptides and adipokines in DIO mice. Three- to four-week-old C57BL/6 J male mice were fed different diets for 16 weeks, the mice were sacrificed and the epididymal fat and intestine were dissected in order to measure mRNA expressions of the genes investigated. (**A–E**) mRNA expressions in the adipose tissue; (**F–I**) mRNA expressions in the colon; (**J–N**) correlation between GPR43 and related genes. n = 8–10 in each group. *Compared to the lean control (**C**) group, P < 0.05; #compared to the HFD group, P < 0.05.

The mRNA levels of GPR43 were significantly lower in the adipose tissue and higher in the colon of the HFD-fed mice compared with those of the control lean mice (P < 0.05) (Fig. 2A and F). Dietary supplementation of acetate, propionate, butyrate or their admixture to the HFD significantly increased the GPR43 expression in the adipose tissue and reduced its expression in the colon (P < 0.05) (Fig. 2A and F). Similar changes to the GPR43 expressions in the adipose tissue and colon were found in the GPR41 mRNA expression in the HFD-fed mice and mice fed the HFD supplemented with SCFAs (Fig. 2B and G). The expression profiles of GPR43 in the liver and muscle were similar to those in the colon, and no differences were found in the expressions of GPR43 and GPR41 in other tissues (hypothalamus, stomach and small intestine) among the six different dietary groups (P > 0.05) (Supplemental Figure S1).

### SCFAs affect expressions of hormone peptides and adipokines in DIO mice

In the HFD-fed mice, the leptin mRNA expression (Fig. 2C) was significantly increased, whereas expressions of adiponectin (Fig. 2D) and resistin (Fig. 2E) in the adipose tissues were decreased compared with those of the lean control mice (P < 0.05). Dietary supplementation of acetate or SCFA admixture in the HFD significantly reduced the leptin mRNA expression (P < 0.05) (Fig. 2C), whereas supplementation of all three SCFAs or their admixture in the HFD significantly increased the mRNA levels of adiponectin and resistin (P < 0.05) (Fig. 2D and E). The mRNA expressions of PYY (Fig. 2H) and GLP-1 (Fig. 2I) in the colon were significantly higher in the HFD-fed mice than those in the lean control mice (P < 0.05). Dietary propionate supplementation to the HFD significantly decreased the PYY mRNA expression, whereas the GLP-1 mRNA expression was reduced by dietary supplementation of acetate, propionate, butyrate or their admixture (P < 0.05) (Fig. 2H and I). Further analysis indicated that the expressions of adiponectin and resistin in the adipose tissues and GLP-1 and PYY in the colon were positively correlated with the mRNA expression of GPR43 (P < 0.05) (Fig. 2K to N). No correlation was found between the expressions of leptin and GPR43 (P > 0.05) (Fig. 2J).

### SCFAs affect fat oxidation in DIO mice

**Figure 3:**
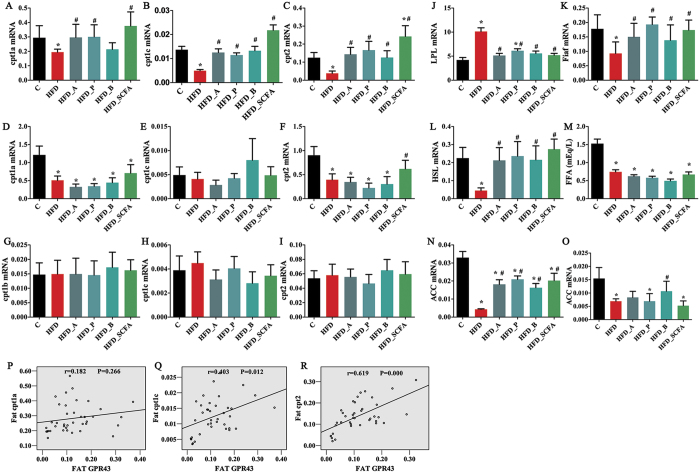
SCFAs regulate lipid metabolism. Three- to four-week-old C57BL/6 J male mice were fed different diets for 16 weeks, the mice were sacrificed and the epididymal fat, liver, intestine and muscle were dissected for measuring mRNA expressions of related genes. Blood samples were harvested by heart puncture and plasma was separated. mRNA expressions of cpt1 and cpt2 in the adipose tissue (**A–C**), liver (**D–F**) and muscle (**G–I**); mRNA expressions of LPL, Fiaf and HSL in the adipose tissue (**J–L**); plasma FFA concentration (**M**); ACC mRNA in the adipose tissue (**N**) and liver (**O**); correlation between fat GRP43 and cpts (**P–R**). n = 8–10 in each group. *Compared to the lean control (**C**) group, P < 0.05; #compared to the HFD group, P < 0.05.

The effects of SCFAs on fat oxidation in obesity are shown in Fig. 3. The mRNA levels of cpt1a, cpt1c and cpt2 in both the adipose tissue (Fig. 3A to C) and the liver (Fig. 3D to F) were decreased significantly in the HFD-fed mice compared to those of the lean control mice (P < 0.05), and this reduction in the adipose tissue but not in the liver was reversed to the levels of the lean control mice by dietary SCFA supplementation (P < 0.05). No differences were found in the expressions of cpt1b, cpt1c and cpt2 in the muscle (Fig. 3G to I) among the six different dietary groups (P > 0.05). Correlative analysis showed a positive relationships between the expressions of cpt1c and cpt2 and GPR 43 expression in the adipose tissue (P < 0.05) (Fig. 3P to R).

The mRNA expression levels of adipose LPL (Fig. 3J) and its regulator, fiaf (Fig. 3K), and HSL (Fig. 3L) were significantly increased and reduced, respectively, in the HFD-fed mice compared to those of the lean control mice (P < 0.05), and dietary SCFA supplementation reversed these changes, resulting in levels similar to those of the lean control mice (P < 0.05). The plasma FFA concentration in the HFD-fed mice was decreased (P < 0.05), and this was not changed by dietary SCFA supplementation (P > 0.05) (Fig. 3M). ACC mRNA expressions in both the adipose tissue (Fig. 3N) and the liver (Fig. 3O) were lower in the HFD-fed mice than those of the control lean mice (P < 0.05), and this reduction was partially reversed by dietary SCFA supplementation in the adipose tissue (P < 0.05), but the levels did not return to normal.

### SCFAs promote beige adipogenesis

**Figure 4:**
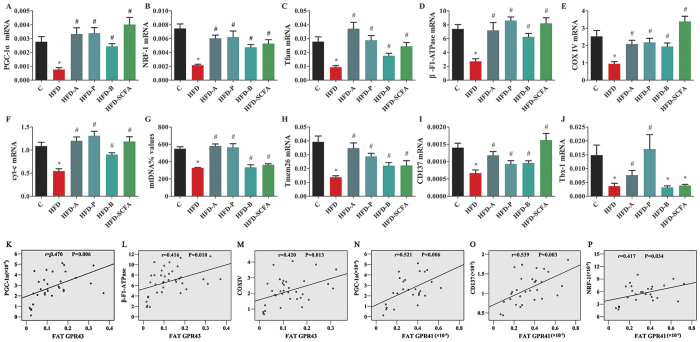
SCFAs regulate beige adipocyte differentiation and mitochondrial biogenesis. Three- to four-week-old C57BL/6 J male mice were fed different diets for 16 weeks, the mice were sacrificed and the epididymal fat was dissected for measuring mRNA expressions of related genes. (**A–F**) Mitochondrial biogenesis-related genes (PGC-1a, NRF-1, Tfam, β-F1-ATPase, COX IV and cyt-c); G: mtDNA quantity; (**H–J**) beige adipocyte markers (Tbx1, Tmem26, CD137). (**K–M**) correlation between fat GRP43 and related genes. n = 8–10 in each group. *Compared to the lean control (**C**) group, P < 0.05; #compared to the HFD group, P < 0.05.

As shown in Fig. 4, expressions of the genes involved in mitochondrial biogenesis (PGC-1a, NRF-1, Tfam, β-F1-ATPase, COX IV and cyt-c) (Fig. 4A to F), mtDNA levels (Fig. 4G) and beige adipocyte markers (Tbx1, Tmem26, CD137) (Fig. 4H to J) were all reduced in the HFD-fed mice compared to those of the lean control mice (P < 0.05), and dietary SCFA supplementation reversed their expressions, resulting in levels close to those of the lean control mice (P < 0.05). Correlative analysis showed that the expressions of PGC-1a, 1β-F1-ATPase and COX IV in the adipose tissue were positively associated with the mRNA expression of GPR43 (P < 0.05) (Fig. 4K to M), and the expressions of PGC-1a, CD137 and NRF1 were positively associated with GPR41 (P < 0.05) (Fig. 4N to P).

### SCFAs promote changes in the gut microbiota

**Figure 5:**
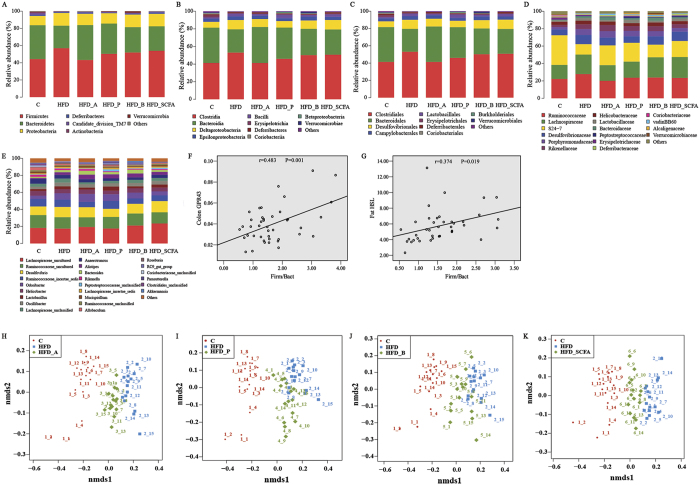
SCFAs alter gut microbiota in DIO mice. (**A–E**) 454 pyrosequencing analysis of the fecal microbiota composition from mice fed different diets at the levels of the phylum, class, order, family and genus. (**H–K**) Bacterial communities based on weighted UniFrac distance. (**F** and **G**) Correlation between the ratio of Firmicutes to Bacteroidetes and the GPR43 expression levels in the adipose tissue and colon. n = 15 in each group.

We generated a dataset of 4,252,385 sequence reads meeting the quality criteria from 90 samples with an average of 507,285 reads per group and 22,007 reads per mouse. Of the reads, 99.99% were assigned at the phylum level, and 98.94%, 98.83%, 98.53% and 72.62% at the class, order, family and genus levels, respectively. Taxonomy-based analysis of the assigned sequences showed that the gut microbiota were dominated by specific species at different levels. Fecal microbial composition of all mice was mostly comprised of Firmicutes (average: 52.27% across all samples) and Bacteroidetes (average: 31.04%) at the phylum level. Other less abundant phyla were Proteobacteria (average: 13.01%), Deferribacteres (average: 1.10%), Actinobacteria (average: 0.82%) and others (Fig. 5A to E). Species richness, coverage and diversity estimations for each data set are shown in Table 2. The bacterial phylotype richness, reflected by the ACE and Chao indexes, was much lower in samples from the HFD-fed mice than that in the control lean mice but could be elevated significantly by adding acetate, propionate, butyrate or their admixture to the diets. The bacterial diversity expressed as Shannon and Simpson indexes did not show any differences among the six dietary groups. Rarefaction and Shannon-Wiener curves for each group indicated that the total bacterial diversity was well represented. More than 99% of coverage in all of the samples indicated that the sequencing depth was sufficient to reflect the whole bacterial diversity and the real composition of gut microbiota (Supplemental Figure S2).

NMDS analysis showed that the identified taxa from the HFD-fed mice, the control lean mice and mice fed the HFD with each type of SCFA were successfully partitioned into three distinct groups (Fig. 5H to K). Analysis using the KW test showed that the microbial clusters from the HFD-fed mice were significantly different from those in the control lean mice, and the clusters in samples from SCFA-containing diet-fed mice were located between the HFD-fed mice and the control lean mice. These data indicate that dietary supplementation of acetate, propionate, butyrate or their admixture partially improved the profile of gut microbiota in the HFD-fed mice.

The gut microbiota composition in the HFD-fed mice shifted at different taxonomic levels from the phylum to the genus and was affected by dietary SCFA supplementation (Fig. 5A to E and Table 3). In the HFD-fed mice, there was an increase in the proportions of Firmicutes and a reduction in the proportions of Bacteroidetes at the phylum level, leading to a higher ratio of Firmicutes to Bacteroidetes (2.17:1) compared with that of the lean mice (1.12:1) (P < 0.05). Several statistical differences were found in low-abundance phyla, indicating that Proteobacteria were higher and Actinobacteria and Candidate_division_TM7 were lower in the HFD-fed mice (P < 0.05). Supplementation of acetate or propionate in the HFD diets significantly reduced and increased the proportions of Firmicutes and Bacteroidetes, respectively (P < 0.05). Correlative analysis showed that the ratio of Firmicutes to Bacteroidetes in feces was positively associated with the GPR43 mRNA expression in the colon and the HSL mRNA expression in the adipose tissue (P < 0.05) (Fig. 5F and G). Different bacterial groups at the genus level showing the effects of SCFA supplementation included *Ruminococcaceae*, *Lachnospiraceae*, *Anaerotruncus* and *Lactobacillus* (Fig. 5A to E and Table 3). Other differences in bacterial abundances suggested that SCFA supplementation to the HFD did not generate a lean-like microbiome.

## Discussion

Although the SCFAs have been shown to protect against body weight gain in both the DIO mice and overweight humans, the underlying mechanisms are unclear. In this study, consistent with previous reports^17^,^24^,^25^,^26^, we demonstrated that dietary supplementation of acetate, propionate, butyrate or their admixture significantly inhibited the body weight gain induced by HFD feeding. Furthermore, these supplemented SCFAs led to significant changes in expressions of GPR43 (an increase in the adipose tissue and a reduction in the colon), as well as bacterial community structure in feces (a reduction in the proportions of Firmicutes and an increase in the proportions of Bacteroidetes) compared to those of the HFD-fed obese mice. SCFAs could modify the structure of the gut microbiota towards a pattern more similar to that of the control mice used in the present study, implying that the alleviation of microbiota disturbance may play a role in SCFA-mediated prevention of obesity.

The production of SCFAs by microbial fermentation of dietary fibers is associated with a wide range of health benefits, including improvements in body composition, energy homeostasis, lipid profiles and reduced body weight, by stimulating multiple hormonal and neural pathways, such as appetite control, enhancement of oxidation and thermogenesis in muscle and liver tissues, and others^6^,^13^,^14^,^15^,^24^,^25^,^26^. However, no consistent conclusions have been reached on changes of gut SCFA production in obesity. It has been demonstrated that fecal SCFA concentrations in rodent models of genetic obesity and overweight adult humans are higher than those of their lean counterparts, indicating an increased microbial energy harvest in obesity^22^,^23^,^27^. However, in diet-induced obese mice fed a HFD with 45% calories from fat, reduced fecal SCFA (acetate and propionate) concentrations have been reported^28^. Unfortunately, in the present study, we did not find any changes in the fecal concentrations of the three types of SCFAs in obese mice fed the HFD with 60% calories from fat. This discrepancy may be the result of different shifts in microbial cross-feeding patterns, diet compositions, severity of obesity, genetic background and others. Interestingly, if SCFAs were added to the HFD, more propionate and butyrate were excreted from the feces with no changes in acetate, suggesting that acetate may be more efficiently absorbed into the body and have greatest effects in preventing weight gain. Regarding the roles of acetate in obesity, Lin HV *et al.* reported less effects of acetate on suppression of excess weight gain compared to propionate and butyrate, and den Besten G *et al.* found that all three SCFAs attenuated body weight to a similar extent^24^,^26^. The inconsistency in the roles of acetate in obesity may be due to differences in mouse species, age, the HFD formula and quantity of SCFAs added. In the former study, three-month-old C57BL/6 N mice were put on HFD (60% of calories from fat) supplemented with molarity-matched sodium salts of butyrate (5% wt wt^−1^), propionate (4.3% wt wt^−1^), or acetate (3.7% wt wt^−1^)^24^; whereas in the latter study, two-month old C57Bl/6 J mice were fed HFD (45% of calories from fat) with sodium acetate, sodium propionate, or sodium butyrate incorporated into the diet at the same ratio of 5% (wt wt^−1^). In our study, the HFD (60% of calories from fat), added acetate, propionate or butyrate at 5% (wt wt^−1^), were fed to three to four week-old C57Bl/6 J mice, and the result showed that all the three SCFAs suppressed the HFD-induced weight gain, with acetate having the greatest effects.

SCFAs have been shown to increase the release of the anorectic gut hormones PYY and GLP-1 from enteroendocrine cells and leptin from adipose tissue, leading to a reduction in food intake and body weight gain^24^,^25^. Various studies that focused on the elucidation of SCFA target molecules in the host indicated that GPR43 and GPR41 are predominantly activated by the SCFAs, with acetate and propionate preferentially activating GPR43 and propionate and butyrate preferentially activating GPR41^15^,^16^. In the present study, the HFD-fed obese mice had higher expressions of GPR43 and GPR41 in the colon, which were positively associated with expressions of PYY and GLP-1. This suggests that higher expressions of GPR 43 and GPR41 in the colon might result in increased expressions of PYY and GLP-1, which may counteract the body weight gain by inhibiting food intake. Dietary supplementation of acetate, propionate, butyrate or their admixture helped to drastically decrease GPR43 and GPR41, thus leading to a reduction in PYY and GLP-1 expressions to keep them within a normal range^29^. The reduction in these anorectic hormones may have prevented decreases in the food and energy intake after introduction of SCFAs to the HFD. Similarly, in the adipose tissue, higher expression of leptin was induced by the HFD and reversed by supplementation with acetate or the SCFA admixture. Regarding the effects of SCFAs on leptin and their relationships with GPRs, a number of contradictory reports have been published. Some studies reported that the SCFAs, propionate and acetate increase the leptin expression in adipocytes *in vitro*, whereas butyrate has no effects^30^,^31^. However, Frost *et al.* failed to demonstrate a significant effect of SCFAs on leptin secretion in adipocytes^32^. Furthermore, the leptin mRNA expression has been found to be up-regulated by GPR43 activation in some studies or by GPR41 activation in others^29^,^30^. Here, we found no correlations between GPR43 and the leptin. These different results reported may be due to the different experimental design (*in vitro* or *in vivo*) and duration, which change the leptin expression^33^. Moreover, the lower expressions of GRP43 in the adipose tissue and associated reductions in adiponectin and resistin were found in the HFD-fed obese mice, and dietary SCFA supplementation significantly recovered their expressions to normal ranges or even higher, leading to reductions in body weight. This is consistent with previous reports indicating that activation of GPR43 in the adipose tissue by SCFAs protects against diet-induced obesity by suppressing insulin signaling as well as increasing the consumption of lipids^17^. The underlying mechanisms may be mediated by SCFA activated expressions of adiponectin and resistin, which have an inverse correlation with severity of insulin resistance^34^,^35^.

Nonetheless, it has been shown that dietary SCFA supplementation increased the expressions of adipose cpt1 and cpt2, the rate-limiting enzymes in mitochondrial fatty acid β-oxidation^36^, which were closely associated with the GPR43 expression herein. Furthermore, we found that SCFA supplementation promoted expressions of the genes associated with mitochondrial biogenesis (PGC-1a, NRF-1, Tfam, β-F1-ATPase, COX IV, cyt-c) and quantity (mtDNA)^37^, suggesting that the regulation of body weight by SCFAs is mediated by promoting mitochondrial function. Beige adipocytes emerging in white fat depots, similar to classical brown adipocytes, are also involved in fat burning. When animals are exposed to cold or receive chronic ß-adrenergic stimulation, the pre-existing beige adipocytes undergo phenotypic transdifferentiation, and browning will appear morphologically and histochemically^38^,^39^. In this study, we found that expressions of several gene markers specific for beige adipocytes were reduced in the HFD-fed obese mice and could be recovered by SCFA supplementation. Furthermore, the expressions of several genes involved in mitochondrial biogenesis and beige adipocytes were positively associated with GPR43 and GPR41 expressions. These data imply that SCFAs may promote beige adipogenesis, resulting in enhanced fat oxidation and energy expenditure, mediated by activation of GPR43 or GPR41. Meanwhile, the increased LPL expression likely resulting from the reduced fiaf expression^19^ along with the decreased HSL expression in fat indicates the inhibition of TG hydrolysis and lower plasma FFA concentration in obese individuals. Supplementation of SCFAs modified the expressions of LPL, fiaf and HSL to normal levels with no changes in plasma FFA concentration, suggesting that more TG was hydrolyzed to release FFAs, which may be oxidized. Thus, the beneficial effects of SCFAs on body weight induced by HFD feeding may primarily depend on their regulation of energy expenditure, presumably by activating GPR43 or brown fat-mediated thermogenesis in fat tissue^17^.

During the past ten decades, a large number of studies demonstrated that the pathogenesis of obesity is closely associated with disturbances in the gut microbiota, and variations in the ratio between the two major intestinal microbial phyla (Firmicutes and Bacteroidetes) have been reported^21^,^40^. However, comparisons of the gut bacterial communities between obese and lean subjects have produced conflicting results. Several studies involving mouse models and humans have shown an increased proportion of Firmicutes and reduced Bacteroidetes in obesity and significant increases in Bacteroidetes along with weight loss^20^,^21^,^22^,^41^,^42^, whereas others have observed no changes in these two phyla or even reported the opposite results^23^,^43^,^44^,^45^. These phylogenetic differences in different reports may be due to many variables, such as diet, genetic background, sample handing, sequencing techniques, and data analysis tools, among others^40^. In the current study, a higher abundance of Firmicutes concomitant with a decrease in the abundance of Bacteroidetes was found in the HFD-fed obese mice, which is consistent with several previous reports^22^,^24^,^41^. Interestingly, dietary supplementation of the SCFAs led to a higher abundance of Bacteroidetes and a reduction of Firmicutes accompanied by significant body weight loss.

It is important to note that most of the above observations were found division-wide, and the development of a microbiome favored by high-fat diets promotes energy harvest and storage-promoting obesity and metabolic diseases. The underlying mechanisms include suppression of the fiaf expression, leading to increased triglyceride storage in adipose tissues by promoting LPL expression^19^,^46^,^47^, and more bacterial lipopolysaccharide (LPS) transferred from the intestinal lumen to the blood, contributing to the low-grade inflammation, reduction in glucose tolerance, body weight gain and fat mass development, and oxidative stress that characterize the metabolic syndrome^47^,^48^. Similar to a previous report^47^, our results showed a significant increase in Proteobacteria, which includes Gram-negative bacteria; this results in increased LPS in the gut, which can act as an inflammatory factor to trigger the onset of insulin resistance, obesity, and diabetes^49^. Accordingly, in this study, the HFD-fed mice had elevated plasma glucose and insulin concentrations as well as increased expressions of plasma IL-1β, IL-6 and MCP-1, which were reduced by dietary SCFA supplementation. Furthermore, SCFAs along with monosaccharides produced by bacterial fermentation in the colon are absorbed and used as substrates for hepatic lipogenesis and gluconeogenesis^20^,^22^, as well as to activate GPR43 and GPR41 in tissues such as the colon and adipose tissue^15^,^16^,^50^. This is supported by our results indicating that the GPR43 expression in the colon was positively correlated with the ratio of Firmicutes to Bacteroidetes, and interestingly, dietary supplementation of the SCFAs down-regulated the ratio of Firmicutes to Bacteroidetes as well as the colonic GPR43 expression. In addition, the reduced ratio of Firmicutes to Bacteroidetes after SCFA supplementation may promote adipose HSL expression, causing increases in the fat hydrolyzed and oxidized. However, these findings need to be corroborated by causative studies in the future.

The shifts in gut microbiota based on obesity at the division level do not always correlate with the shifts at lower taxonomic levels. Sequencing in this current study showed that the HFD-fed obese mice had higher abundances of the genera *Bacteroides, Odoribacter* and *Alistipes*, and dietary SCFA supplementation did not change their abundance or increase them further. In addition, differences in taxa abundance at the phylum level have little relevance when considering all their individual groups^21^,^40^. Thus, many bacterial groups at lower taxonomic levels deserve attention, such as the genera *Desulfovibrio* in the *Proteobacteria* phylum, which may be linked to obesity^51^ and can be reduced by glycomacropeptide application^52^. Accordingly, in this current study we showed that the HFD-fed obese mice had a higher *Desulfovibrio* abundance, and adding acetate and propionate to diets reduced its abundance, but this did not reach statistical significance. Additionally, the abundance of *Ruminococcaceae* in the *Firmicutes* phylum, a family containing several butyrate-producing bacteria, was found to be higher^53^ or lower^54^ in obese mice. In post-surgery individuals, the fecal microbiota was characterized by an overall decrease of bacterial diversity, with a significant reduction in *Ruminococcaceae*, *Lachnospiraceae*, *Clostridiaceae*, *Eubacteriaceae*, and *Coriobacteriaceae*^55^. Intestinal colonization by *Lachnospiraceae*, which belongs to *Firmicutes*, isolated from the feces of hyperglycemic obese mice contributes to the development of diabetes in obese mice^56^. With regard to these bacteria, we found higher abundances of *Ruminococcaceae_incertae_sedis* and *_unclassified*, *Lachnospiraceae_incertae_sedis* and *_unclassified* in the DIO mice, and their reduction was associated with SCFA supplementation to some extent. The genera *Lactobacillus* and *Bifidobacterium* containing probiotic strains are linked to body weight. In the HFD-fed obese mice, the level of *Bifidobacterium* is reduced, whereas prebiotics promote a selective increase of this genus, and this increase is correlated with reduced adiposity and microbe-derived inflammation^57^. In contrast, higher concentrations of *Lactobacillus* species belonging to the Firmicutes phylum were found in obesity both in rodents and humans in comparison with lean subjects^58^,^59^. Consistent with these reports, we found that *Lactobacillus* abundance in the HFD-fed obese mice as well as obesity in preschool-age children (data not shown) was increased and that addition of acetate or butyrate reduced the level of *Lactobacillus* in mice fed a HFD. The changes in *Bifidobacterium* among different groups are difficult to explain because of very low abundance of *Bifidobacterium* detected in our study, and this needs to be investigated further.

## Conclusions

In summary, dietary SCFA supplementation prevents the body weight gain induced by high-fat diet feeding, and this may be associated with the regulation of GPR expression, beige adipogenesis, mitochondrial biogenesis and gut microbiota compositions, which finally result in enhanced TG hydrolysis and FFA oxidation in the adipose tissue and inhibited chronic inflammation. These findings provide insights into new targeting mechanisms of SCFAs, which may be important for preventing or treating obesity.

## Methods

### Diets

Five types of high-fat diet (34.9% fat by wt., 60% kcal)—a control high-fat diet (HFD) and four high-fat diets with acetate (HFD-A), propionate (HFD-P), butyrate (HFD-B) and their admixture (HFD-SCFA)—were designed according to the high-fat diet formula (D12492) for the DIO mice from Research Diets, Inc. (New Brunswick, NJ). These five HFD contained the same amount of lard and soy oil as the main source of fat in each diet. Sodium acetate, sodium propionate, sodium butyrate (Sigma-Aldrich, St. Louis, MO) or their admixture (ratio at 3:1:1) was incorporated into the diet at a proportion of 5% (wt wt^−1^)^26^. In addition, a low-fat diet (4.3% fat by wt., 10% kcal) was also designed as a lean control with lard and soy oil as the source of fat, based on the control diet formula (D12450B) from Research Diets, Inc. The diets were prepared by the Institute of Laboratory Animal Sciences at the Chinese Academy of Medical Sciences and stored at −20 °C before use.

### Animals

Three- to four-week-old C57BL/6 J male mice were purchased from the SPF Laboratory Animal Technology Co., Ltd. (Beijing) and housed at the animal facilities under a 12 hour (h) light 12 h^−1^ dark cycle with cycles of air ventilation and free access to water and food in the Laboratory Animal Center of the Academy of Military Medical Sciences of China. After one week of recovery from transportation, the mice were fed one of the five HFD or the control diet at 17:00 each day to induce obesity, and the body weights were measured weekly for 16 weeks. The diet intake was detected at 2, 8, and 12 weeks after feeding. At the end of the experiments, fresh stool samples were collected and stored at −80 °C. Meanwhile, the 12 h fasted mice (n = 10 in each group) were anesthetized by intraperitoneal injection of Avertin (125 mg^ ^kg^−1^ of 2,2,2-tribromoethanol, T-4840–2, Sigma-Aldrich Chemie GmbH, Steinheim, Germany) to obtain blood samples by heart puncture. The plasma was stored at −80 °C for later analysis of biochemical parameters. After sacrifice, the mouse epididymal fat, liver and femoral muscle were dissected free of the surrounding tissue, removed, wrapped in aluminum foil and frozen in liquid N_2_. Once an entire group of animals was harvested, the tissues were transferred to −80 °C until the analysis was performed.

All of the animal experiments were performed from 08:00 to 12:00 in accordance with the recommendations in the Guide for the Care and Use of Laboratory Animals of National Administration Regulations on Laboratory Animals of China. All experimental protocols (No.2014–08-bch) were approved by the Committee on the Ethics of Animal Experiments of the Academy of Military Medical Sciences.

### Analysis of biochemical parameters

Plasma triglyceride (TG) and total cholesterol concentrations were assayed by an enzymatic procedure (GPO-PAP) and the COD-CE-PAP method using a triacylglycerol kit and total cholesterol assay kit, respectively, and plasma glucose concentration was determined by the GOD-PAP method with a glucose assay kit (Sichuan MAKER Science Tech. Co., Ltd., China). Measurements of the plasma free fatty acids (FFA) and insulin concentrations were performed using a NEFA kit (Japan, Wako, 294–63601) and an insulin ELISA kit (Nanjing Jiancheng Bioengineering Institute, China). Examinations of plasma IL-1β, IL-6, IL-10, MCP-1 and TNF-α were performed using a mouse IL-1beta Platinum ELISA kit (BMS614/2), a mouse IL-6 Platinum ELISA kit (BMS603/2), a mouse IL-10 Platinum ELISA kit (BMS6002), a mouse MCP-1 Platinum ELISA kit (BMS6005) and a mouse TNF-alpha Platinum ELISA kit (BMS607/3) (eBioscience Inc. San Diego, CA, USA).

### Gene expression: RNA extraction from tissues and quantification by RT-PCR

Total RNA was extracted from mouse epididymal fat, liver, brain, intestine and muscle using TRIzol Reagent (cat. no. 15596–206, Invitrogen, Carlsbad, CA, USA), and the cDNA was reverse transcribed from the total RNA using the SuperScript^TM^ III First-Strand Synthesis System for RT-PCR (cat. no. 18080–051, Invitrogen, Carlsbad, CA, USA) according to the procedures provided by the manufacturer. The GPR41 and GPR43 mRNA levels in the above tissues were measured using real-time quantitative RT-PCR with an ABI PRISM 7300 system (Applied Biosystems, Foster City, CA, USA). Additionally, the expression levels of target genes involved in lipogenesis (acetyl-CoA carboxylase, ACC), triglyceride hydrolysis and lipolysis (hormone sensitive lipase, HSL; lipoprotein lipase, LPL) and energy expenditure (carnitine palmitoyltransferase-1 and 2, cpt1 and cpt2), mitochondrial biogenesis (peroxisome proliferator–activated receptor-γ coactivator 1a,PGC-1a; nuclear respiratory factor 1, NRF-1; mitochondrial transcription factor A, Tfam; b subunit of the mitochondrial H+-ATP synthase, β-F1-ATPase; cytochrome c oxidase IV, COX IV; cytochrome c somatic, cyt-c), beige adipocyte markers (T-box 1, Tbx1; transmembrane protein 26, Tmem26; tumor necrosis factor receptor superfamily, member 9, CD137) were determined^37^,^38^,^39^,^60^. The oligonucleotide primers for the targeted genes in this study were designed and tested for efficiency using Primer-BLAST (http://www.ncbi.nlm.nih.gov/tools/primer-blast/) (Supplemental Table S1). The co-amplification of β-actin or 18 S mRNA, invariant internal controls, was performed in all of the samples. The assays were performed in triplicate, and the results were normalized to the internal standard mRNA levels using the 2^−ΔCT^ method. Mitochondrial DNA (mtDNA) copy number was determined by qPCR analysis of the cytochrome B (cytB) mtDNA gene compared with the large ribosomal protein p0 (36B4) nuclear gene, as previously described^61^.

### Fecal SCFA detection: gas chromatographic analysis

Fecal SCFAs were measured using gas chromatography (GC) according to a previously described method^62^ with some modifications. Specifically, 150 mg of each fresh stool sample was weighed, suspended in sterile distilled water (1.2 ml) and homogenized for approximately 3 min. Then, the pH of the suspension was adjusted to 2–3 by adding 5 M HCl, and the sample was incubated at room temperature for 10 min with occasional shaking. The suspension was transferred into a polypropylene tube and centrifuged for 10 min at 14,000 rpm, and the centrifugation steps were repeated until the supernatant was clear. The internal standard, 2-ethylbutyric acid solution, was spiked into the supernatant at a final concentration of 1 mM, and the supernatant was injected in an Agilent 6890 N GC system equipped with a flame ionization detector (FID) and an N10149 automatic liquid sampler (Agilent, USA). A high-resolution gas chromatography column (DB-624UI, J&W Scientific, Agilent Technologies Inc., USA) of 30 m × 0.25 mm i.d. coated with 1.40 μm film thickness was used. Nitrogen was supplied as the carrier gas at a flow rate of 1.1 mL/min. The initial oven temperature was 60 °C, maintained for 1.0 min, raised to 160 °C at 10 °C/min and held for 2.0 min, then increased to 200 °C at 5 °C/min, and finally held at 200 °C for 3 min. The temperatures of the FID and the injection port were 220. The flow rates of hydrogen, air and nitrogen as makeup gas were 30, 350 and 45 mL/min, respectively. The injected sample volume for GC analysis was 1 μL, and the run time for each analysis was 23.5 min.

### Microbial community analysis: extraction of DNA from fecal samples and 16 S rRNA sequencing using MiSeq technology

Microbial DNA was extracted from mouse feces using a QIAamp DNA Stool Mini Kit (cat. no. 51504, Qiagen, Germany) according to the manufacture’s protocol. DNA extraction was assessed by agarose gel electrophoresis. The V3-V4 region of the bacteria 16 S ribosomal RNA gene was amplified by PCR (95 °C for 5 min, followed by 25 cycles at 95 °C for 30 s, 60 °C for 30 s, and 72 °C for 25 s and a final extension at 72 °C for 5 min) using primers 338 F 5′-ACTCCTACGGGAGGCAGCA-3′ and 806 R 5′-GGACTACHVGGGTWTCTAAT-3′. Barcodes that allow sample multiplexing during pyrosequencing were incorporated between the 454 adaptor and the forward primer. PCR amplification was performed in a 20 μL reaction system using TransGen AP221–02: TransStart FastPfu DNA Polymerase (TransGen Biotech, China) with an ABI GeneAmp^®^ 9700 sequence detection system (ABI, Foster City, CA, USA). The PCR products were purified using an AxyPrep DNA Gel Extraction Kit (AXYGEN, USA) and then mixed equally before pyrosequencing. The PCR products of the V3–V4 region of the 16 S rRNA gene were sequenced by the Next Generation Sequencing Core using an Illumina MiSeq PE300^63^. The data have been submitted to the SRA database and will be accessible with the following link after the indicated release date: http://www.ncbi.nlm.nih.gov/sra/SRP078781 (SRA accession: SRP078781; temporary Submission ID: SUB1682690).

### Microbial community analysis: biodiversity analysis and phylogenetic taxonomy

To obtain high-quality sequences, the raw sequences were filtered and trimmed as previously reported^64^,^65^,^66^. Then, sequences were clustered into operational taxonomic units (OTUs) with a distance limit of 0.03 (equivalent to 97% similarity) using the Usearch software (version 7.1 http://drive5.com/uparse/). According to the cluster information, Rarefaction and Shannon-Wiener curves, the Chao and Ace estimator for community richness (http://www.mothur.org/wiki/Chao, http://www.mothur.org/wiki/Ace), the Shannon and Simpson index for community diversity (http://www.mothur.org/wiki/Shannon, http://www.mothur.org/wiki/Simpson), and the Good’s coverage for sequencing depth (http://www.mothur.org/wiki/Coverage) were assessed for each sample using Mothur software (version v1.30.1, http://www.mothur.org/wiki/Schloss_SOP#Alpha_diversity). Taxonomical assignments of OTUs were performed using Mothur software in accordance with the SILVA database (release 119 http://www.arb-silva.de) at an 80% confidence level. Finally, the sequences were phylogenetically assigned to taxonomic classifications using an RDP Classifier (version 2.2 http://sourceforge.net/projects/rdp-classifier/) at a 70% confidence level. After phylogenetic allocation of the sequences down to the phylum, class, order, family and genus levels, the relative abundance of a given phylogenetic group was defined as the number of sequences affiliated with that group divided by the total number of sequences per sample.

To clarify the similarities of fecal microbiota between the experimental groups, Venn diagrams and species rank abundance distribution curves (Whittaker plots) were generated using R-project for statistical computing. In addition, non-metric multidimensional scaling (NMDS) and hierarchical cluster analysis were performed to determine whether the OTUs identified using the KW filter discriminate between different groups by examining relationships between ecological communities, such as those of the microbiota^67^,^68^. Meanwhile, the taxonomy of the fecal microbiota was assessed by a taxon-dependent analysis using the RDP classifier to identify specific bacteria associated with SCFAs.

### Statistical analysis

One-way analysis of variance (ANOVA) was performed to compare means in different groups with normally distributed data using SPSS version 13.0 for Windows; for data with a non-normal distribution, the differences between groups were assessed using the Mann–Whitney U-test and the Wilcoxon signed-rank test. Significant differences between the lean control group and the HFD-fed obese group were assessed using Dunnett’s test, and comparisons between the HFD-fed obese group and groups supplemented with different SCFAs were determined by the SNK test. All values are expressed as the mean ± SD, and P < 0.05 was considered to be statistically significant.

## Additional Information

**How to cite this article**: Lu, Y. *et al.* Short Chain Fatty Acids Prevent High-fat-diet-induced Obesity in Mice by Regulating G Protein-coupled Receptors and Gut Microbiota. *Sci. Rep.* **6**, 37589; doi: 10.1038/srep37589 (2016).

**Publisher's note:** Springer Nature remains neutral with regard to jurisdictional claims in published maps and institutional affiliations.

## Supplementary Material

Supplementary Information

## Figures and Tables

**Table 1:** Effects of SCFAs on biochemical parameters and fecal SCFA excretion in DIO mice.

	C	HFD	HFD_A	HFD_P	HFD_B	HFD_SCFA
Plasma						
Glucose (mmol/L)	5.26 ± 0.86	10.61 ± 2.52*	9.50 ± 2.13*	11.29 ± 2.10*	9.27 ± 2.79*	11.43 ± 1.78*
Triglyceride (mmol/L)	0.60 ± 0.07	0.78 ± 0.08*	0.55 ± 0.09^#^	0.62 ± 0.08^#^	0.63 ± 0.09^#^	0.60 ± 0.10^#^
Total cholesterol (mmol/L)	2.12 ± 0.57	3.44 ± 0.25*	1.78 ± 0.47^#^	2.85 ± 0.80*	1.93 ± 0.64^#^	2.47 ± 0.37^#^
Insulin (mIU/L)	8.50 ± 0.51	9.56 ± 0.82*	9.01 ± 1.00	10.92 ± 0.58*	10.69 ± 0.33*	9.89 ± 0.92
FFAs (mEq/L)	1.52 ± 0.31	0.74 ± 0.17*	0.62 ± 0.12*	0.58 ± 0.12*	0.49 ± 0.15*	0.67 ± 0.21*
IL-1β (ng/L)	3.99 ± 0.25	4.99 ± 0.46*	3.79 ± 0.53^#^	4.07 ± 0.59	4.38 ± 0.85	3.87 ± 0.44^#^
IL-6 (ng/L)	48.91 ± 8.89	75.39 ± 28.09*	47.24 ± 7.60^#^	43.32 ± 12.35^#^	50.44 ± 8.26^#^	48.23 ± 9.74^#^
IL-10 (ng/L)	10.29 ± 1.43	11.42 ± 1.52	12.87 ± 1.81*	12.73 ± 1.53*	12.72 ± 1.02*	13.48 ± 1.57*^#^
MCP-1 (ng/L)	80.05 ± 15.95	106.81 ± 18.56*	105.36 ± 16.23*	85.14 ± 18.17^#^	98.67 ± 19.79	81.81 ± 13.22^#^
TNF-α (ng/L)	15.10 ± 3.55	18.27 ± 5.63	14.34 ± 3.39	14.25 ± 2.82	10.10 ± 1.65^#^	12.57 ± 3.11
Feces						
Acetate (mg/g)	14.68 ± 2.99	12.90 ± 2.75	11.22 ± 2.90	10.68 ± 3.17	10.53 ± 2.16	13.11 ± 4.27
Propionate (mg/g)	2.85 ± 0.94	2.17 ± 0.68	1.93 ± 0.63	5.13 ± 1.60*^#^	2.18 ± 0.60	2.98 ± 1.00
Butyrate (mg/g)	1.92 ± 0.55	1.21 ± 0.67	0.98 ± 0.34	1.27 ± 0.54	2.67 ± 1.62^#^	1.70 ± 0.57

Note: n = 8–10 in each group. Data are means ± SD. *Compared to the lean control (C) group, P < 0.05; ^#^Compared to the HFD group, P < 0.05.

**Table 2:** Characteristics of sequences in different groups.

Groups	sequences				97% similarity			
	valid	normalization	OUT	coverage	ACE	Chao	Shannon	Simpson
C	500,489	330,105	5740	99.74 ± 0.03	422.87 ± 20.52	423.33 ± 20.37	4.26 ± 0.26	0.04 ± 0.02
HFD	510,398	330,105	5057*	99.75 ± 0.03	380.33 ± 19.02*	385.27 ± 17.74*	4.31 ± 0.17	0.03 ± 0.02
HFD_A	467,275	330,105	5453*^#^	99.74 ± 0.03	406.87 ± 20.65^#^	410.53 ± 20.18^#^	4.46 ± 0.14	0.02 ± 0.01
HFD_P	482,945	330,105	5280*^#^	99.74 ± 0.04	396.67 ± 24.69*^#^	399.67 ± 26.38*^#^	4.39 ± 0.19	0.03 ± 0.01
HFD_B	516,012	330,105	5393*^#^	99.74 ± 0.03	403.67 ± 27.51 ^#^	411.60 ± 30.25^#^	4.39 ± 0.21	0.03 ± 0.01
HFD_SCFA	558,494	330,105	5695^#^	99.73 ± 0.03	424.33 ± 14.69 ^#^	432.60 ± 18.50^#^	4.41 ± 0.19	0.03 ± 0.01

Note: the number of OTUs, coverage percentages, richness estimators (ACE and Chao), and diversity indices (Shannon and Simpson) were calculated at 3% distance. n = 15 in each group. Data are means ± SD. *Compared to the lean control (C) group, P < 0.05; ^#^Compared to the HFD group, P < 0.05.

**Table 3:** Taxonomic classification of pyrosequences from bacterial communities at the phylum and genus levels.

Group	C	HFD	HFD_A	HFD_P	HFD_B	HFD_SCFA
**Firmicutes**	44.27 ± 1.82	56.91 ± 2.45*	43.26 ± 1.90^#^	50.38 ± 1.59*^#^	51.94 ± 2.10*	53.96 ± 1.68*
*Lachnospiraceae_uncultured*	11.66 ± 0.98	15.16 ± 1.26*	14.47 ± 1.92	13.49 ± 1.14	17.48 ± 1.18*	18.75 ± 1.82*
*Ruminococcaceae_uncultured*	9.61 ± 0.81	11.61 ± 0.76*	8.44 ± 0.47^#^	10.34 ± 0.73	11.73 ± 0.82	10.37 ± 0.55
*Ruminococcaceae_incertae_sedis*	5.53 ± 0.61	7.38 ± 0.75*	4.71 ± 0.26^#^	7.12 ± 0.47*	5.19 ± 0.47^#^	5.32 ± 0.48^#^
*Lachnospiraceae_incertae_sedis*	0.79 ± 0.07	1.28 ± 0.09*	0.71 ± 0.06^#^	1.22 ± 0.11*	1.15 ± 0.14*	1.09 ± 0.07*
*Lachnospiraceae_unclassified*	2.22 ± 0.27	4.07 ± 0.36*	1.67 ± 0.13^#^	2.19 ± 0.20^#^	3.05 ± 0.19*^#^	2.82 ± 0.23^#^
*Lachnospiraceae_uncultured*	11.66 ± 0.98	15.16 ± 1.26*	14.47 ± 1.92	13.49 ± 1.14	17.48 ± 1.18*	18.75 ± 1.82*
*Anaerotruncus*	3.59 ± 0.40	3.72 ± 0.38	2.79 ± 0.19^#^	2.24 ± 0.21*	2.43 ± 0.17*^#^	3.02 ± 0.19
*Allobaculum*	2.01 ± 0.62	1.14 ± 0.35	0.44 ± 0.12*	0.93 ± 0.28	0.30 ± 0.10*^#^	0.71 ± 0.19
*Oscillibacter*	1.86 ± 0.16	3.91 ± 0.51*	2.91 ± 0.33*	2.87 ± 0.36*	3.25 ± 0.31*	3.34 ± 0.36*
*Lactobacillus*	1.37 ± 0.20	2.87 ± 0.69	1.72 ± 0.35^#^	3.74 ± 0.69*	1.88 ± 0.42^#^	2.78 ± 0.62*
**Bacteroidetes**	39.39 ± 2.56	26.21 ± 2.83*	40.54 ± 2.22^#^	35.13 ± 1.89^#^	29.46 ± 2.78*	28.33 ± 2.34*
*Odoribacter*	2.57 ± 0.32	6.56 ± 0.63*	6.87 ± 0.50*	6.00 ± 0.82*	5.70 ± 0.79*	3.79 ± 0.29*^#^
*Rikenella*	1.57 ± 0.22	1.63 ± 0.20	1.61 ± 0.16	0.74 ± 0.09*^#^	0.93 ± 0.13*^#^	1.02 ± 0.13*^#^
*Bacteroides*	0.93 ± 0.19	2.16 ± 0.33*	3.36 ± 0.38*^#^	1.97 ± 0.36*	3.71 ± 0.71*	1.70 ± 0.33
*Alistipes*	0.66 ± 0.10	2.69 ± 0.31*	4.01 ± 0.44*^#^	3.71 ± 0.43*	3.77 ± 0.51*	2.70 ± 0.32*
**Proteobacteria**	10.78 ± 0.80	14.75 ± 1.53*	13.19 ± 0.75*	12.06 ± 0.83	14.63 ± 1.59*	14.60 ± 1.41*
*Desulfovibrio*	6.49 ± 0.56	10.44 ± 1.24*	8.89 ± 0.64*	7.35 ± 0.70	9.44 ± 1.43	10.58 ± 1.01*
*Helicobacter*	3.49 ± 0.03	4.09 ± 0.04	3.86 ± 0.03	3.25 ± 0.03	4.26 ± 0.04	3.55 ± 0.03
**Candidate_division_TM7**	1.76 ± 0.25	0.67 ± 0.08*	0.81 ± 0.11*	0.80 ± 0.10*	1.15 ± 0.19^#^	1.02 ± 0.11*^#^
**Actinobacteria**	1.40 ± 0.26	0.32 ± 0.10*	0.09 ± 0.01*^#^	0.38 ± 0.16*	0.22 ± 0.08*	0.11 ± 0.01*
**Deferribacteres**	1.25 ± 0.22	0.86 ± 0.16	1.42 ± 0.38	0.59 ± 0.16*	0.79 ± 0.18	1.53 ± 0.44
*Mucispirillum*	1.25 ± 0.22	0.86 ± 0.16	1.42 ± 0.38	0.59 ± 0.16*	0.79 ± 0.18	1.53 ± 0.44

Note: n = 15 in each group. Data are shown as the mean ± SD. *Compared to the lean control (C) group, P < 0.05; ^#^Compared to the HFD group, P < 0.05.
