# Supplementary material for: Short Chain Fatty Acids Prevent High-fat-diet-induced Obesity in Mice by Regulating G Protein-coupled Receptors and Gut Microbiota
Source: Sci Rep. 2016 Nov 28;6:37589. doi: 10.1038/srep37589 (PMC5124860; doi:10.1038/srep37589)
Supplement: Supplementary Information [file srep37589-s1.doc]

**Supplementary Information**

Short Chain Fatty Acids Prevent High-fat-diet-induced Obesity in Mice by Regulating G Protein-coupled Receptors and Gut Microbiota

Yuanyuan Lu, Chaonan Fan, Ping Li, Yanfei Lu, Xuelian Chang, Kemin Qi*

**Table S1. The PT-PCR primers for the targeted genes in this study**

| **Primer** | **Forward primer (5'to3')** | **Reverse primer (5'to3')** |
| --- | --- | --- |
| GPR43 | ACAGTGGAGGGGACCAAGAT | GGGGACTCTCTACTCGGTGA |
| GPR41 | TTGCTAAACCTGACCATTTCGG | GATAGGCCACGCTCAGAAAAC |
| GLP-1 | CAAACCAAGATCACTGACAAGAAAT | GGGTTACACAATGCTAGAGGGA |
| PYY | CTTCACAGACGACAGCGACA | GGGAAATGAACACACACAGCC |
| Fiaf | CACCCACTTACACAGGCCG | GAAGTCCACAGAGCCGTTCA |
| HSL | ATGCCACTCACCTCTGATCC | CTGTCCTGTCCTTCCCGTAG |
| Resistin | CCTGCTAAGTCCTCTGCCAC | GGCTTCATCGATGGGACACA |
| Aiponectin | TGACGACACCAAAAGGGCTC | ACCTGCACAAGTTCCCTTGG |
| Leptin | TGGCTTTGGTCCTATCTGTC | TCCTGGTGACAATGGTCTTG |
| LPL | TGAAAGCCGGAGAGACTCAG | AGTGTCAGCCAGACTTCTTCAG |
| Cpt1a | TTTGAATCGGCTCCTAATGG | CCCAAGTATCCACAGGGTCA |
| Cpt1b | CATGTATCGCCGCAAACTGG | CCTGGGATGCGTGTAGTGTT |
| Cpt1c | CCCCAATACCCCTACATCCT | ATCCCCGATACCCCTGTCT |
| CPT2 | ACCACAACATCCTGTCCACC | GTGGAGAAACTCTCGGGCAT |
| ACC | CGAAGGGCTTACATTGCCTA | GGATGTTCCCTCTGTTTGGA |
| PGC-1α | AGCCGTGACCACTGACAACGAG | GCTGCATGGTTCTGAGTGCTAAG |
| Nrf1 | CAAGTCCAGCAGGTCCATGT | GTTACCTCATCAGCTGCCGT |
| Tfam | AAGAACGCATGGAGGAGAGA | TTCTGGGGAGAGTTGCAGTT |
| β-F1-ATPase | CGTGAGGGCAATGATTTATACCAT | TCCTGGTCTCTGAAGTATTCAGCAA |
| COX IV | TTAACGAGAGCTTCGCCGAG | CCAAATCAGAACGAGCGCAG |
| Cyt-c | ATAGGGGCATGTCACCTCAAAC | GTGGTTAGCCATGACCTGAAAG |
| Tmem26 | ACCCTGTCATCCCACAGAG | TGTTTGGTGGAGTCCTAAGGTC |
| CD137 | CGTGCAGAACTCCTGTGATAAC | GTCCACCTATGCTGGAGAAGG |
| Tbx1 | GGCAGGCAGACGAATGTTC | TTGTCATCTACGGGCACAAAG |
| Cyt B | TCCTACTGGTCCGATTCCAC | ATGTCGTTTTGGGTGAGAGC |
| 36B4 | AGGATATGGGATTCGGTCTCTTC | TCATCCTGCTTAAGTGAACAAACT |
| β-actin | GGCCAACCGTGAAAAGATGA | CAGCCTGGATGGCTACGTACA |
| 18S | AGGATGTGAAGGATGGGAAG | TTCTTCAGCCTCTCCAGGTC |


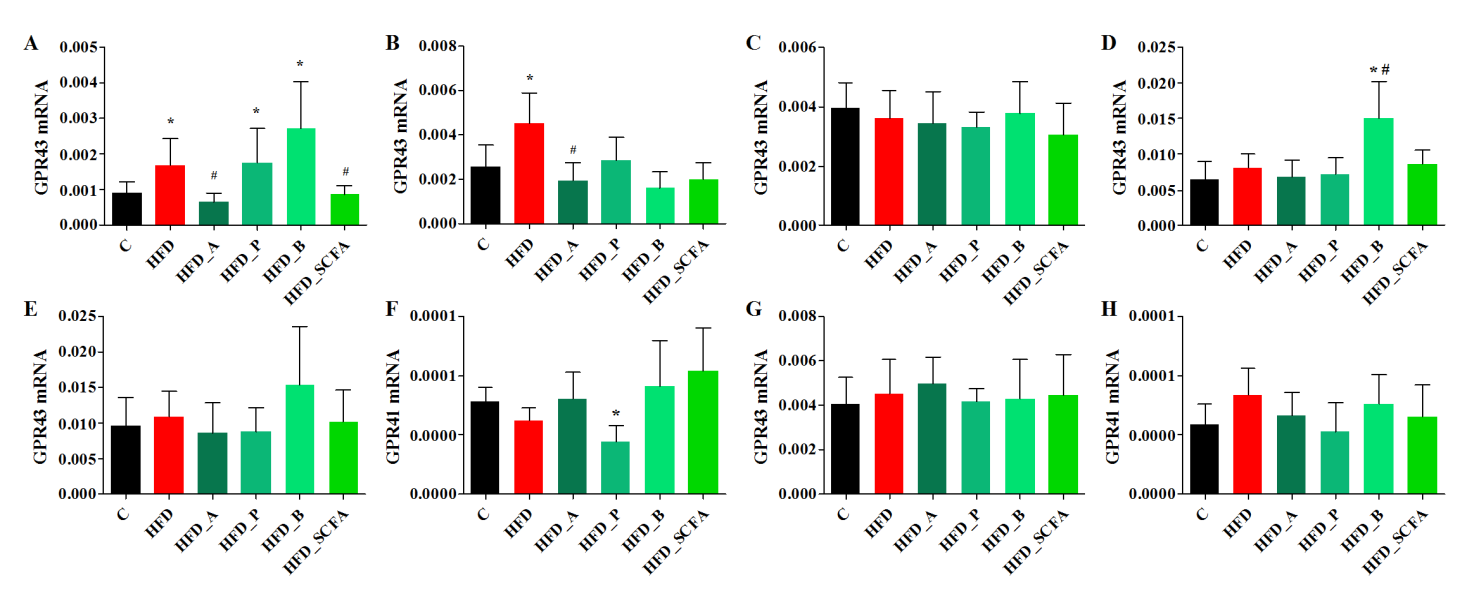


**Figure S1. SCFAs affected the expressional profiles of GRP43, GPR41 in the other tissues of DIO mice.** A-D: mRNA expressions in the liver, muscle, hypothalamus and stomach respectively. E and F: mRNA expressions in the jejunum. G and H: mRNA expressions in the ileum. n=8-10 in each group. * Compared to the lean control (C) group, P<0.05; # Compared to the HFD group, P<0.05.


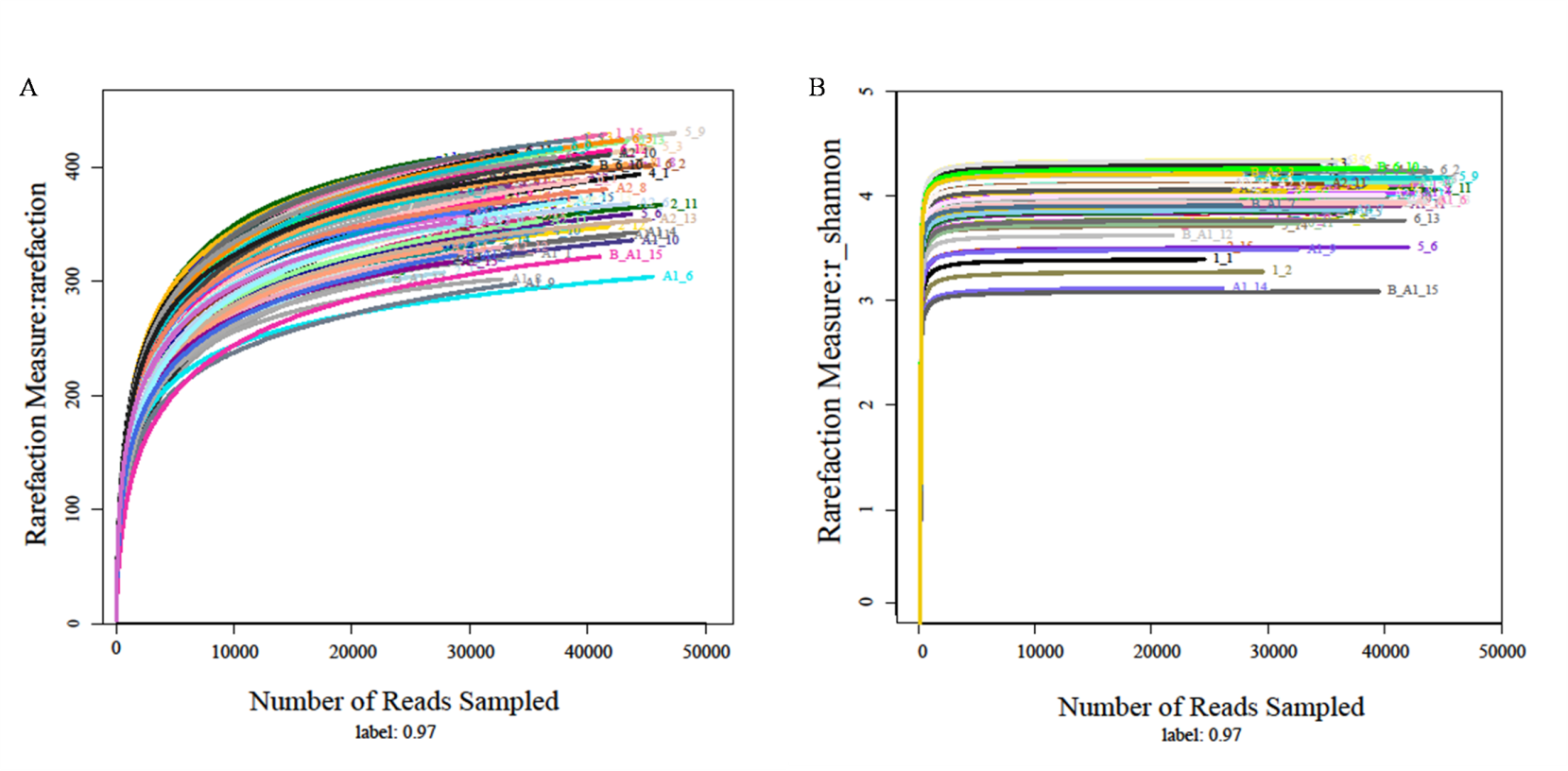


**Figure S2. Rarefaction and Shannon-Wiener curves of bacterial 16S rRNA sequences for the samples.** OTUs are identified using 97 % cutoffs. A: Rarefaction curve reflects sequencing depth; B Shannon-Wiener curve helps to decide validity of gut microbiota composition.
